# Subacute Bacterial Endocarditis with* Leptotrichia goodfellowii* in a Patient with a Valvular Allograft: A Case Report and Review of the Literature

**DOI:** 10.1155/2016/3051212

**Published:** 2016-11-08

**Authors:** Wilfredo R. Matias, Daniel L. Bourque, Tomoko Niwano, Andrew B. Onderdonk, Joel T. Katz

**Affiliations:** ^1^Harvard Medical School, Boston, MA, USA; ^2^Division of Infectious Diseases, Massachusetts General Hospital, Boston, MA, USA; ^3^Tokyo Medical and Dental University, Tokyo, Japan; ^4^Department of Pathology, Brigham and Women's Hospital, Boston, MA, USA; ^5^Division of Infectious Diseases, Brigham and Women's Hospital, Boston, MA, USA

## Abstract

*Leptotrichia *species are normal constituents of the oral cavity and the genitourinary tract microbiota that are known to provoke disease in immunocompromised patients and rarely in immunocompetent individuals. Following the description of* Leptotrichia goodfellowii *sp. nov., two cases of endocarditis by this species have been reported. Here, we report a case of* Leptotrichia goodfellowii* endocarditis in an immunocompetent patient with a valvular allograft. The isolation and identification of* Leptotrichia* can be challenging, and it is likely that infection with this pathogen is significantly underdiagnosed. A definitive identification, as in this case, most often requires 16S rRNA gene sequencing, highlighting the increasingly important role of this diagnostic modality among immunocompetent patients with undetermined anaerobic bacteremia.

## 1. Introduction

*Leptotrichia* species are normal constituents of the oral cavity [1], intestinal tract [2], and genitourinary tract [3] that produce lactic acid as a major metabolic end product [4]. Although part of the resident microbiota,* Leptotrichia *species can spread from primary colonization sites and are known to cause infections in humans [5]. Outside of the oral cavity, they have been associated with aortic aneurysms [6] and hepatic abscesses [7] and are known to cause bacteremia in immunocompromised hosts [8]. However, they are rarely recovered from the blood of patients who are not immunocompromised; the few reported cases of infection in apparently immunocompetent hosts were cases of endocarditis in patients with preexisting cardiac valvular lesions [9–11]. Here, we report the third definitive case of* L. goodfellowii* endocarditis in an immunocompetent patient with a cardiac lesion, identified by 16S rRNA gene sequencing.

## 2. Case Report

A 44-year-old male with a history notable for a bicuspid aortic valve that was repaired 19 years ago via a Ross procedure (pulmonary autograft replacement of the aortic valve and cadaveric allograft replacement of the pulmonary valve) was admitted for evaluation of 2-3 months of persistent daily fevers up to 39.4°C, headaches, night sweats, and marked fatigue of unknown origin. Review of systems was remarkable for a week of mild painless hematochezia. He was using aspirin frequently for control of his fevers. His family and social history were unremarkable beyond owning a pet dog. He reported a dental cleaning approximately 5 months prior to presentation. At the time of presentation, he had undergone extensive infectious and rheumatologic evaluations for persistent but undifferentiated fevers. Blood cultures obtained three months prior to admission, and again one week prior to admission at a separate facility in Bactec Plus aerobic and anaerobic FAN bottles, processed in a Bactec 9120 system (Becton Dickinson), were negative after 5 days of incubation.

On physical examination the patient was febrile to 38.8°C. He was diaphoretic but otherwise well appearing. He had no evidence of dental or periodontal disease and no visible oropharyngeal abnormalities. Cardiovascular examination was notable for an old III/VI holosystolic murmur greatest at the left lower sternal border, radiating to the back, and an old II/VI diastolic murmur best appreciated over the RUSB. The pulmonary exam was unremarkable. Abdominal exam was notable for slight tenderness to palpation in the RLQ. On rectal exam, no perianal lesions were noted and there were no masses in the rectal vault; he had brown, guaiac positive stools but no melena. No other findings of endocarditis were appreciated.

Laboratory evaluation revealed a hematocrit that was decreased to 30.7% from a preillness baseline of 48%, an erythrocyte sedimentation rate (ESR) of 44 mm/hr, C-reactive protein (CRP) of 55 mg/L, and creatinine of 1.01 mg/dL. His white blood cell count was 6,000 per mcL. A urinalysis with sediment was notable for 1+ blood, and 2 red blood cells per high-powered field on microscopy. Transthoracic and transesophageal echocardiography revealed mild, diffuse thickening of the mitral valve with trace mitral regurgitation and focal thickening of the pulmonic valve homograft, which were highly suggestive of a valvular vegetation. There was stable, severe dilation of the aortic root. These findings were confirmed with cardiac magnetic resonance imaging.

Blood cultures were sent on the day of admission and processed with the BacT/ALERT system (bioMerieux) and on day 2 grew thin, tapered Gram-variable rods from an anaerobic FAN bottle. Subcultures grew only on* Brucella* agar plates incubated within an anaerobic chamber, and Gram stain of the culture revealed thin Gram-negative rods with tapered ends. This isolate was negative for oxidase and spot indole testing. The patient was started on vancomycin, ceftriaxone, and metronidazole for empiric coverage. Multiple blood cultures continued to be positive for Gram-negative rods from anaerobic FAN bottles, and Gram-variable rods were also subsequently isolated from both aerobic and anaerobic FAN bottles over the following 2 days during which the patient was taking antibiotics. Despite the positive blood cultures, the patient defervesced and felt better following the administration of antibiotics. Blood cultures drawn on day 3 of antibiotic treatment were negative and remained negative after 5 days. At discharge, the isolate was still not identified.

Additional work-up with gas-liquid chromatography revealed a large peak for lactic acid and a small peak for acetic acid. The appearance on Gram stain, aerotolerance noted on further passage, and lactic acid production were most consistent with* Leptotrichia* species. A RapID ANAII test, which uses conventional and chromogenic substrates for species identification, did not reveal any satisfactory matches. Isolates were sent for 16S rRNA sequencing as a definitive diagnosis was unattainable via conventional phenotypic methods. Following 16S rRNA sequencing, a search of the Isentio RipSeq 16S database yielded a match with a 99.9% identity for* L. goodfellowii* and 7.3% difference separating it from another related species.

The patient was discharged on piperacillin/tazobactam and metronidazole as these antibiotics had demonstrated clinical effectiveness in a previous case report [9]. Antibiotic susceptibility testing of the isolates via Etest showed sensitivity to ceftriaxone (MIC 0.016 *μ*g/mL), clindamycin (MIC 0.016 *μ*g/mL), metronidazole (MIC 0.25 *μ*g/mL), and penicillin (MIC 0.125 *μ*g/mL). Following the availability of susceptibility data, the patient was switched to ceftriaxone and metronidazole and in total completed a 6-week course of antibiotics. He was then started on chronic suppressive therapy with penicillin V potassium, which was complicated by elevated liver transaminases and discontinued. He was then started on cefpodoxime, which he has tolerated well. Cardiac surgery was under consideration but ultimately deferred as he has been otherwise asymptomatic and with stable cardiac imaging since his hospitalization.

## 3. Discussion

We present an uncommon case of endocarditis with* Leptotrichia goodfellowii* in an immunocompetent patient with a history of a Ross procedure. Our patient met 1 major and 2 minor Duke criteria for possible infective endocarditis (positive blood cultures, fever, and predisposing condition). A diagnosis of infective endocarditis was further supported by his chronic night sweats, fevers, and elevated inflammatory markers (ESR and CRP). Given the likelihood of infective endocarditis, and his predisposing cardiac risk factors, he was promptly started on antibiotic treatment following identification of positive blood cultures.

*Leptotrichia* are a rare cause of human infections, with only 54 cases described in the literature as of 2008 [5]. Given the difficulty in isolating and identifying these organisms using currently available methods, it is possible that their role in human disease is underestimated. A 62-month retrospective survey of 4,857 episodes of anaerobic bacteremia identified* Leptotrichia *spp. as the causative pathogen in 7.3% of cases [12]. With the advent of improved diagnostics and sequencing technology, it is possible that their designation as emerging pathogens in human disease will likely be further elucidated. The first two documented cases of* L. goodfellowii* infection were isolated from blood cultures of patients with endocarditis [9]. Subsequently, this species was isolated from blood cultures obtained from a stillborn thought to have expired secondary to fetal bacteremia [13], wound cultures from a patient with a wound infection following a dog bite, and most recently from an immunocompetent patient with bacteremia [14], suggesting that this organism has broader infectious potential than previously appreciated [15].

The association between* Leptotrichia* species and endocarditis is rare. Our case accounts for the fifth reported case of* Leptotrichia* species endocarditis (Table 1). Three of these cases were definitively identified as* Leptotrichia goodfellowii* [9], and another was due to a* Leptotrichia buccalis*-like organism [10] that was likely* L. goodfellowii*, possibly suggesting a link between this particular species and endocarditis. Additionally, whereas the majority of infections with* Leptotrichia* sp. have been documented in immunocompromised hosts, none of the cases of endocarditis occurred in immunocompromised patients, suggesting that preexisting cardiac lesions are on their own risk factors for developing infection with this organism. Infection in our patient presented in a subacute fashion, unlike three of the four previously reported cases, which had more fulminant courses.* Leptotrichia* sp. are predominantly susceptible to beta-lactam antibiotics, carbapenems, clindamycin, chloramphenicol, tetracyclines, and metronidazole but resistant to vancomycin and aminoglycosides [2]. Our patient successfully completed a 6-week course of targeted antibiotic therapy following definitive identification, which was followed by chronic suppressive therapy.

*Leptotrichia* species are difficult to isolate and identify [16]. It may take several days for a culture to grow. If isolated, it can be difficult to distinguish from closely related organisms such as* Fusobacterium*,* Capnocytophaga*, and* Lactobacillus*. They produce lactic acid as a major metabolic end product, differentiating them from other filamentous bacteria such as* Fusobacterium* and* Bacteroides* [5]. They are normally straight or curved, non-spore forming, nonmotile rods with one or both ends pointed or rounded [17]; they often demonstrate variability in their appearance on Gram staining. Although they normally grow under anaerobic conditions, they can also develop aerotolerance following repeated culture at 37°C in the presence of CO_2_ [5]. As demonstrated in this case, conventional phenotyping methods are seldom adequate, making accurate diagnosis mainly dependent on molecular methods. The causative organism in our case was predictably difficult to isolate and identify. Notably, blood cultures when the patient was symptomatic on two occasions prior to admission did not grow the organism. It is unclear why no growth was observed on multiple occasions prior to admission, whereas all blood cultures performed at our hospital demonstrated growth. Cultures prior to the current admission were performed using Bactec Plus aerobic and anaerobic FAN bottles and processed in a Bactec 9120 system (Becton Dickinson) system, whereas cultures performed at our hospital were processed using the BacT/ALERT system (bioMerieux). Both FA and FAN bottles used in our system are enriched and contain charcoal to neutralize antibiotics and other inhibitory substances. Once isolated at our institution, identification via conventional methods was inconclusive and ultimately required the use of 16S rRNA sequencing technology.

Although rare, this case should alert clinicians about the potential pathogenicity of* Leptotrichia* species, as they can cause endocarditis in patients with preexisting cardiac conditions. Given the difficulty in isolation and identification, this pathogen should also be considered in cases of blood culture negative endocarditis, and the threshold for utilizing 16S rRNA sequencing at the first signs of difficulty isolating and identifying the causative organism should be low. This case also demonstrates the utility of 16S rRNA sequencing for the identification of difficult to identify pathogens. Decreasing time to identification can reduce delays in implementing the appropriate antibiotics and lead to better outcomes. Finally, further research into the pathogenicity of this species would allow us to better appreciate its pathogenic potential and better identify and treat infections caused by this organism.

## Figures and Tables

**Table 1:** Reported cases of *Leptotrichia* sp. endocarditis.

Reference	Year	Patient age	Sex	Presentation	Predisposing cardiac condition	Valve affected	Definitive identification method	Organism isolated	Antibiotic regimen	Surgical intervention	Outcome
Current case	2015	44	M	Subacute endocarditis	Bioprosthetic pulmonic valve, aortic valve homograft	Pulmonic	16S rRNA sequencing	*L. goodfellowii*	(1) Vancomycin, ceftriaxone, metronidazole (2) Piperacillin/tazobactam, metronidazole (3) Ceftriaxone, metronidazole	No	Recovery
Caram et al.	2008	74	F	Subacute endocarditis	Mitral regurgitation	Mitral	16S rRNA sequencing	*L. goodfellowii*	Piperacillin/tazobactam	Yes	Mitral valve replacement with complete resolution of symptoms
Caram et al.	2008	52	M	Acute endocarditis	Bioprosthetic aortic valve	Aortic	16S rRNA sequencing	*L. goodfellowii*	Ceftriaxone, metronidazole	Yes	Recovery
Hammann et al.	1993	65	F	Subacute endocarditis	Prosthetic aortic valve	Aortic	(1) Anaerobic Schaedler agar with 5% sheep blood, Columbia agar in air with 5% CO_2_ (GasPak CO_2_ system, BD) (2) Conventional tests for fermentation reactions and gas chromatography	*L. buccalis*-like	(1) Cefotaxime, metronidazole (2) Vancomycin, imipenem	Yes	Recovery and surgery, followed by deterioration and death secondary to acute LV failure and anterior MI
Duperval et al.	1984	24	M	Subacute endocarditis	Endocardial cushion defect, coarctation of the aorta, Down Syndrome	Mitral	Gas chromatography after growth on peptone-yeast-glucose agar	*L. buccalis*	Penicillin G	No	Recovery
